# Nonconvulsive Status Epilepticus Complicating Epstein-Barr Virus Encephalitis in a Child

**DOI:** 10.1155/2014/547396

**Published:** 2014-03-12

**Authors:** Filippo Greco, Maria Donatella Cocuzza, Pierluigi Smilari, Giovanni Sorge, Lorenzo Pavone

**Affiliations:** ^1^Unit of Clinical Pediatrics, Department of Medical and Pediatric Sciences, University of Catania, Via Santa Sofia, 95123 Catania, Italy; ^2^Unit of Child Neuropsychiatry, Department of Medical and Pediatric Sciences, University of Catania, Catania, Italy; ^3^Unit of Pediatrics and Pediatric Emergency, Azienda Ospedaliera-Universitaria O.V.E. Policlinico, University of Catania, Catania, Italy

## Abstract

Children with acute encephalopathy show prolonged electrographic seizure activity consistent with nonconvulsive status epilepticus (NCSE). Pediatric NCSE is a heterogeneous clinical entity with poor outcome and different etiologies, including central nervous system infection, stroke, toxic-metabolic syndrome, and epileptic syndrome. We report a 4-year-old girl with seizure and behavioral changes in whom the analysis of cerebrospinal fluid by polymerase chain reaction was positive for Epstein-Barr virus. We emphasize the importance of electroencephalography (EEG), and particularly, of continuous EEG monitoring for early recognition and appropriate treatment of this condition.

## 1. Introduction

Epstein-Barr virus (EBV) is associated in childhood with many neurological manifestations, including encephalitis, meningitis, myelitis, cerebellitis, cranial nerves palsy, acute disseminated encephalomyelitis, and acute inflammatory polyneuropathy (Guillain-Barré syndrome; GBS) [1].

We report a 4-year-old girl with seizures and confusional state, followed by an electroencephalography (EEG) pattern consistent with nonconvulsive status epilepticus (NCSE), where EBV was detected by polymerase chain reaction performed on cerebrospinal fluid.

## 2. Case Presentation

The patient was the third female child born to nonconsanguineous parents. The family history was unremarkable. She was born at 38 weeks gestation by normal delivery after uncomplicated pregnancy. Birth weight was 3.2 kg, length 49 cm, and head circumference 34 cm. The perinatal period was uneventful and psychomotor development was normal.

At the age of 3 years and 11 months, the patient developed fever and sore throat lasting for 3 days. A diagnosis of bacterial tonsillitis was made and treatment with oral cephalosporin was started. After 1 month, in good health, she exhibited a motor partial complex seizure involving arm and left leg lasting for less than 5 minutes and followed by an acute confusional state that lasted for 3 hours.

At admission to the Clinical Pediatric Division of the University of Catania, her weight was 14 kg (10th percentile), height 96 cm (10th percentile), and head circumference 50 cm (50th percentile). On clinical examination she was not febrile and a mild cervical lymphadenopathy was evident. Cardiovascular and respiratory physical exam including blood pressure were normal and the liver and spleen were within normal limits. Irritability and significant behavioral changes, in particular, in arousal and memory were observed. Mild hyposthenia of the upper limbs was evident; there were no signs of meningeal irritation, and cranial nerves exam was normal, as were ocular fundoscopy and pupillary reflexes. Muscle trophism, perception of touch and temperature, and deep tendon reflexes were all normal.

**Figure 1:**
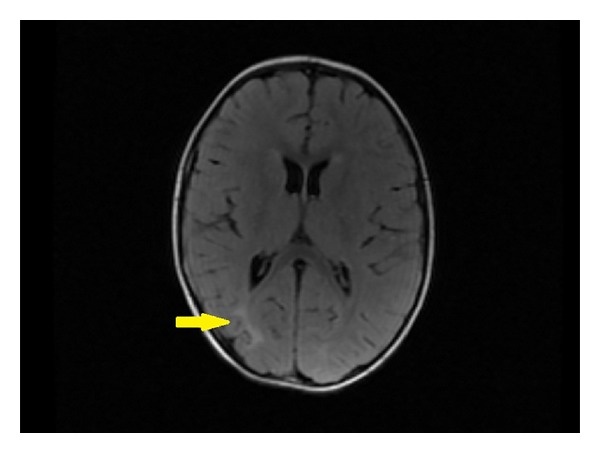
Brain MRI showed a subcortical increased signal in the right occipital lobe on T2-weighted image.

**Figure 2:**
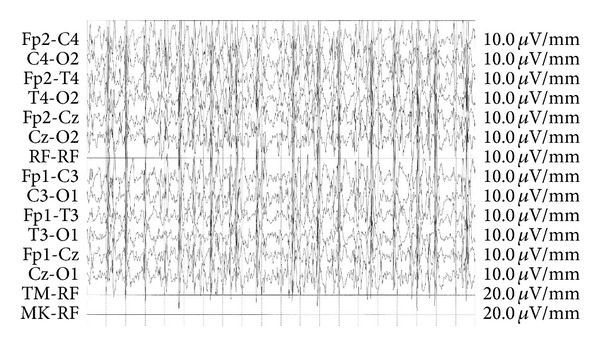
EEG showed a generalized slow activity with high amplitude. Spikes and spikes-slow waves complex appear singly or in group and are unilateral or bilateral. When bilateral, they can occur synchronously or independently. The parameters used for EEG registration are sens: 10 microV/mm, TC: 0,10 s, and HF: 15,0 Hz. The montage is longitudinal type.

Laboratory evaluation revealed a normal leukocyte count (10 860/*μ*L), normal erythrocyte sedimentation rate (13 mm/hour), and normal serum C-reactive protein level (0,1 mg/dL). Magnetic resonance imaging (MRI) of the brain and spinal cord revealed a subcortical increased signal in the right occipital lobe (Figure 1). EEG monitoring performed was a surface routine EEG lasting for 30 minutes; it showed disorganization in the interval with the presence of a chaotic structuring of background activity, low-voltage spiking, and primary generalization, which indicated a diagnosis of NCSE (Figure 2). Treatment with orally sodium valproate (20 mg/kg/day) was started in the first day; we made an EEG every day during the period of acute phase and an improvement of EEG patterns, particularly of organization of background activity, was observed after three days.

The following clinical investigations were all within the normal range: chest radiography, urinalysis, red blood cell count, platelet count, glucose, serum urea, serum electrolytes, transaminases, bleeding time, fibrinogen, immunoglobulins, and antibodies to* Mycoplasma pneumoniae*,* Toxoplasma gondii*, HIV,* Borrelia burgdorferi*, and cytomegalovirus. The IgM antibody titers against EBV were 4,71 UI/mL (0–0.9) and IgG antibody titers were 8.2 UI/mL (0.7–35), and IgG antibody titers against EBV early antigen were also positive at 1 : 160, suggesting acute EBV infection. CSF examination showed mild pleocytosis (20 cells/mm^3^ (all lymphocytes)), with normal glucose and protein concentrations. EBV DNA was detected in the CSF with a nested PCR using 30 cycles and various DNA viruses (cytomegalovirus, herpes simplex virus (HSV), varicella-zoster virus (VZV), and enterovirus) were negative. Therefore, acyclovir was initiated at a dose of 10 mg/kg//day intravenously in the third day. After about three days of admission, patient showed a progressive improvement of general conditions and in particular of behavioral changes and mental status.

One month after treatment, she presented a full clinical recovery and neurological examination was normal. EEG showed normal activity with a good *α* organization in the occipital area. A second brain MRI performed 10 months later was unchanged and demonstrated the same findings in the right occipital lobe. Two years after onset, EEG was normal and treatment with sodium valproate was slowly discharged.

## 3. Discussion

Acute encephalopathy is a heterogeneous clinical syndrome of reduced consciousness associated with infectious diseases, metabolic disorders, drugs, and other factors. Encephalitis is defined as inflammation of the brain parenchyma. It can be caused by a postinfectious condition such as in acute disseminated encephalomyelitis (ADEM), or to an infective process, which is diffuse and usually viral. HSV-1, VZV, EBV, mumps virus, measles virus, and enteroviruses are responsible for most cases of viral encephalitis in immunocompetent individuals [2].

The diagnosis of EBV-associated encephalitis in the reported patient was made according to clinical and serological findings, electroencephalographic features, subcortical gray matter involvement on MRI, and, above all, the detection of viral DNA on cerebrospinal fluid.

Various CNS manifestations of EBV infections have been documented, such as Bell's palsy, hypoglossal nerve palsy, optic neuritis, chronic fatigue syndrome, GBS, transverse myelitis, aseptic meningitis, and encephalitis [1, 3]. In immunocompetent patients, encephalitis mainly occurs as a short-term complication of mononucleosis, up to 3 weeks after onset, and less frequently after viral reactivation.

The symptoms of EBV encephalitis are heterogeneous in the pediatric age group and include fever, headache, nausea, vomiting, altered consciousness, seizures, meningism, and focal neurological signs. The subtle presentation is with low-grade fever, behavioral changes, language disturbance, and psychosis. A special form of EBV encephalopathy is “Alice in Wonderland syndrome,” which is characterized by visual hallucinations and perceptual distortions [4, 5]. The pathogenesis of EBV encephalitis is not fully understood and is considered by several authors to be consistent with an immune-mediated process rather than direct invasion of the brain [6]. The most frequently areas are the cerebral hemispheres, basal ganglia, cerebellum, brain stem, thalamus, and limbic system. Favorable prognostic criteria are considered hemispheric purely with gray matter or white matter involvement. On the contrary, thalamus and limbic system localization is associated with a higher degree of sequelae [7]. The patient reported here was affected by seizures, irritability, and behavioral changes consistent with acute encephalopathy. To the best of our knowledge, she is the youngest immunocompetent child to be reported with NCSE, in which EBV was detected in CSF by PCR. With respect to pathogenic mechanism, the presence of virus DNA in the CSF implies a primary infection, with localization of occipital gray matter and not reactivation of a previous infection.

Another interesting aspect was the clinical onset with seizures and behavioral changes, and especially, EEG patterns consistent with NCSE. NCSE is often underdiagnosed in children and is defined as a cognitive or behavioral change that lasts for at least 30 min, with evidence of seizures on EEG [8]. In childhood it includes a range of different conditions that can be classified into three groups: acute neurological injuries (encephalitis, stroke, metabolic syndromes, trauma, and intoxication); epileptic syndromes (Dravet syndrome, Lennox-Gastaut syndrome, and myoclonic astatic syndrome); and nonprogressive encephalopathy (Angelman syndrome and Landau-Kleffner syndrome). NCSE includes also “Panayiotopoulos syndrome,” which sometimes encloses only an “autonomic status epilepticus” [9] without other associated symptoms and that rarely an “unrecognized” ESES condition (associated or not with some epileptic syndromes, such as the “Landau-Kleffner” syndrome) could be responsible for “sleep” and “cognitive” impairment in epileptic children [10].

In the first group, NCSE is associated with a high incidence of coma or stupor at the time of diagnosis, as well as a poor outcome [11]. However, it may occur in the absence of any acute etiology, which can make it difficult to perform correct and early recognition. In a clinical and EEG study performed in 19 pediatric patients with NCSE, the majority of patients had preceding seizures and most of these seizures were brief and isolated convulsions, rather than convulsive status epilepticus (CSE) [11].

In the presence of undefined altered mental status or of significant cognitive and behavioral change often following convulsive seizures, routine EEG, and where possible continuous EEG, should be considered, therefore playing a major role in the management of critically ill children.

In a recent study of 100 critically ill children with acute encephalopathy, it was observed that continuous EEG led to specific clinical management changes in 59 children. The most effects were anticonvulsive agent initiation, escalation, or discontinuation [12] and performance of urgent neuroimaging.

In conclusion, the present case emphasizes the need to evaluate the role of EBV in many acute neurological syndromes in childhood, especially in presence of significant and persistent behavioral changes with EEG evidence of seizures. We recommend routine and continuous EEG in critically ill children for early recognition and management of NCSE. Further clinical observations and long-term prospective studies are necessary to improve management and outcome of children affected.

## Abbreviations

NCSE: Nonconvulsive status epilepticus
EEG: Electroencephalography
EBV: Epstein-Barr virus
GBS: Guillain-Barrè syndrome
MRI: Magnetic resonance imaging
PCR: Polymerase chain reaction
CSF: Cerebrospinal fluid
HSV: Herpes simplex virus
VZV: Varicella-zoster virus
ADEM: Acute disseminated encephalomyelitis
CSE: Convulsive status epilepticus.
